# A Duplex Isothermal RPA–Lateral Flow Platform for Concurrent Detection of *Staphylococcus Aureus* and mecA-Mediated Methicillin Resistance

**DOI:** 10.3390/diagnostics16203295

**Published:** 2026-10-10

**Authors:** Jutturong Ckumdee, Somchai Santiwatanakul, Kwanchai Onruang, Thongchai Kaewphinit

**Affiliations:** 1 Department of Clinical Pathology, Faculty of Medicine, Vajira Hospital, Navamindradhiraj University, Bangkok 10300, Thailand; jutturong@nmu.ac.th; 2 Department of Pathology, Faculty of Medicine, Srinakharinwirot University, Nakhon Nayok 26120, Thailand; titi41@yahoo.com (S.S.); kwanchai@g.swu.ac.th (K.O.); 3 Center of Excellence in Medical and Environmental Innovation Research (CEMEIR), Srinakharinwirot University, Bangkok 10110, Thailand; 4 Innovative Learning Center, Srinakharinwirot University, Bangkok 10110, Thailand

**Keywords:** recombinase polymerase amplification, methicillin-resistant *Staphylococcus aureus*, duplex isothermal amplification, lateral flow dipstick, nasal colonization screening

## Abstract

**Background/Objectives:** Rapid identification of *Staphylococcus aureus* and differentiation of methicillin-resistant *S. aureus* (MRSA) remains critical to infection control and antimicrobial stewardship, particularly where rapid screening is desirable. In this study, we developed and evaluated a duplex recombinase polymerase amplification coupled with lateral flow dipstick (RPA–LFD) platform enabling simultaneous detection of the species-associated *spa* locus and the methicillin resistance determinant *mecA* within a single isothermal reaction. **Methods:** Primer–probe architecture and reaction parameters were systematically adjusted, with 39 °C selected as the operating temperature, to enable concurrent visual detection of both targets within 15 min. **Results:** In a preliminary ten-fold serial dilution experiment, visible detection was observed down to 10^1^ CFU/mL for both targets, whereas conventional PCR produced visible amplification down to 10^2^ CFU/mL under the respective assay conditions. This difference represents an assay-specific observation and was not used to establish a statistically validated detection limit. No cross-reactivity was observed against a panel of non-target bacterial species. A preliminary evaluation of 25 nasal swab-derived DNA specimens showed complete concordance with reference PCR within this limited sample set. **Conclusions:** These findings demonstrate the feasibility of concurrent visual detection of species-associated and resistance targets within a single RPA–LFD configuration. Although further analytical and clinical validation is required, the platform provides a technical framework for simplified duplex isothermal detection of *S. aureus* and *mecA*.

## 1. Introduction

*S. aureus* (SA) and methicillin-resistant *S. aureus* (MRSA) are common pathogens that cause community and healthcare-associated infections [1]. MRSA represents a major contributor to the global burden of drug-resistant bacterial infections [2]. Methicillin resistance is driven by acquisition of *mecA*, which encodes the low-affinity penicillin-binding protein PBP2a and enables continued peptidoglycan cross-linking in the presence of β-lactam antibiotics [3]. Therefore, accurate differentiation between *S. aureus* and MRSA is essential for appropriate antimicrobial selection and infection control decision-making [4]. Although culture-based workflows remain the reference standard, their 48–72 h turnaround time constrains rapid intervention [5]. PCR-based assays reduce diagnostic delay and provide high analytical accuracy [4], but dependence on thermocycling instrumentation, stable electrical infrastructure, and trained personnel limits broader deployment, particularly in decentralized settings [6]. These operational constraints have driven continued interest in isothermal amplification platforms that operate at constant temperature within compact diagnostic formats.

Several isothermal nucleic acid amplification strategies, including loop-mediated isothermal amplification (LAMP), helicase-dependent amplification (HDA), rolling circle amplification (RCA), and recombinase polymerase amplification (RPA), have been developed to enable molecular diagnostics under simplified thermal conditions [7,8,9]. Among these, RPA operates at low and constant temperatures (approximately 37–42 °C) and employs a recombinase-mediated strand invasion mechanism that enables primer targeting without thermal denaturation. Amplification typically proceeds within 20–30 min under isothermal conditions, supporting integration with lateral flow detection (LFD) and other portable readout systems [10,11]. RPA-based assays have been applied to diverse bacterial and viral pathogens and are increasingly explored in decentralized and field-adapted diagnostic configurations [12,13].

However, multiplex implementation within recombinase-driven systems introduces additional biochemical considerations. Because RPA proceeds under a single thermal condition, reaction dynamics are influenced by potential competition among primer sets for binding to recombinase protein, which can suppress amplification of one target in favor of another [11]. Efficient strand invasion depends on sufficient primer–template complementarity, particularly within the 3′ anchor region, where mismatches can markedly impair reaction kinetics and reduce the probability of amplification success [14]. In duplex or multiplex configurations, differences in amplicon length and primer design characteristics may influence relative amplification performance between targets [11,14]. From a platform engineering perspective, maintaining balanced amplification efficiency between targets therefore requires deliberate optimization of primer architecture and reaction parameters to minimize competitive bias and preserve categorical interpretability at the detection interface.

These reaction-level challenges are directly relevant to MRSA screening. Detection of *mecA* alone may be misleading, as the gene is also present in coagulase-negative staphylococci and exhibits structural variability within Staphylococcal cassette chromosome mec (SCCmec) elements [4]. Incorporating a species-specific *S. aureus* marker alongside *mecA* in a single reaction enhances interpretive reliability and reduces the likelihood of misattribution of resistance determinants. While this multi-target strategy has been explored through various molecular approaches, such as magnetic bead-assisted PCR, graphene oxide-based mfLAMP–LFA, and RPA-PfAgo detection systems, their evaluations have largely relied on laboratory-prepared samples or defined clinical isolates [15,16,17]. Consequently, reports describing streamlined duplex RPA–LFD configurations tailored specifically for direct nasal colonization screening remain limited.

The nasal cavity serves as a primary site of *S. aureus* colonization, with asymptomatic carriers representing a significant risk factor for subsequent invasive infection [18,19]. Comprehensive reviews of staphylococcal nasal carriage have consistently shown that a substantial proportion of otherwise healthy individuals harbor *S. aureus* in the anterior nares, with MRSA representing a clinically significant subset across community and occupational settings [20]. Meta-analysis evidence further demonstrates that MRSA colonization status carries meaningful negative predictive value for subsequent infection, particularly in low- to moderate-prevalence settings where negative predictive values can exceed 90% [21]. A prospective evaluation suggests that rapid MRSA nasal PCR screening can reduce unnecessary anti-MRSA therapy without increasing mortality or compromising patient safety [22]. Collectively, these findings support the need for timely, decentralized screening tools capable of informing infection prevention and antimicrobial stewardship practices.

Taken together, these considerations highlight the need for an instrument-minimal platform capable of concurrently identifying *S. aureus* and its methicillin resistance determinant within a single reaction. Implementation of this functionality within an RPA–LFD framework requires assay configurations capable of supporting concurrent detection of both targets under constant-temperature conditions. The *spa* locus has been incorporated into multiplex and rapid molecular diagnostic platforms for species-level identification of *S. aureus*, reflecting its practical utility as a species-associated genetic target [23,24]. Although rare deletions within this locus have been reported in specific clonal lineages, these observations further emphasize the importance of deliberate primer design and assay configuration in spa-based detection strategies [24]. In the present study, we developed and analytically evaluated a streamlined duplex RPA–LFD platform enabling simultaneous detection of the *spa* locus and *mecA* within a unified isothermal reaction. Primer–probe configurations, reaction temperature, and incubation parameters were systematically optimized to enable concurrent visual detection of both targets, and analytical performance was assessed alongside clinical concordance using nasal swab-derived specimens. Unlike previously reported MRSA-oriented RPA approaches, the present assay integrates the species-associated spa locus and the methicillin-resistance determinant *mecA* within a single duplex RPA reaction coupled directly to a visually interpreted lateral-flow readout. The principal contribution of this study is therefore the development and preliminary evaluation of this specific *spa*/*mecA* duplex configuration for concurrent species confirmation and resistance-marker detection, rather than the introduction of RPA–LFD itself.

## 2. Materials and Methods

### 2.1. Bacterial Strains and DNA Preparation

Reference strains of *S. aureus* and MRSA were obtained from the HRH Princess Maha Chakri Sirindhorn Medical Center. Identification and antimicrobial susceptibility testing were performed using the VITEK 2 system (bioMérieux, Hazelwood, MO, USA) with GP and AST-P592 panels. The AST-P592 panel includes oxacillin (0.25–4.0 µg/mL) for minimum inhibitory concentration (MIC) determination and a cefoxitin (6 µg/mL) screening test, in accordance with Clinical and Laboratory Standards Institute (CLSI) susceptibility breakpoint criteria. The entire testing process required approximately 24–72 h to complete.

The MRSA reference strain was confirmed by detection of the staphylococcal cassette SCCmec gene complex using the Xpert MRSA NxG assay (Cepheid, Sunnyvale, CA, USA). Isolates stored at −70 °C were subcultured on sheep blood agar and incubated at 35 °C for 18–24 h. A single colony was subsequently subcultured once more before use. DNA was extracted from serial dilutions prepared from a colony suspension adjusted to a turbidity of 0.5 McFarland using a DensiCHEK Plus instrument (bioMérieux, Durham, NC, USA), corresponding to approximately 1.5 × 10^8^ colony-forming units (CFU)/mL at a wavelength of 580 nm.

Each DNA sample from ten-fold (1:10) serial dilutions, ranging from 10^6^ to 10^0^, was processed using the manufacturer’s magnetic bead-based nucleic acid extraction kit (Zybio, Chongqing, China). The dilution range was subsequently confirmed using standard plate count agar (Difco & BBL, Franklin Lakes, NJ, USA). Briefly, 1 mL of each dilution was centrifuged at 10,000× *g* for 2 min, and the supernatant was reduced to 100 µL. Subsequently, 100 µL of pre-treatment solution was added, and the mixture was vortexed. The suspension was incubated at 90 °C for 10 min, then cooled to room temperature for approximately 2 min before processing using the Zybio EXM3000 instrument (Zybio, Chongqing, China).

### 2.2. Ethical Approval and Clinical Specimens

This study was approved by the Human Research Ethics Committee of Srinakharinwirot University (Approval No. SWUEC 210/58). All clinical specimens were anonymized before laboratory access to protect patient privacy and confidentiality. No personal identifiers were available to the investigators during sample processing, laboratory testing, or data analysis. Genomic DNA from previously cultured bacterial isolates, including *S. aureus*, MRSA, *Streptococcus pneumoniae*, *Klebsiella pneumoniae*, *Escherichia coli*, *Mycobacterium tuberculosis*, *Staphylococcus epidermidis*, *Streptococcus mitis*, *Streptococcus mutans*, *Salmonella enterica*, *Lactobacillus paracasei*, *Actinomyces naeslundii*, *Aggregatibacter actinomycetemcomitans*, *Prevotella intermedia*, and *Porphyromonas gingivalis*, was obtained from the Department of Pathology, Faculty of Medicine, HRH Princess Maha Chakri Sirindhorn Medical Center, Srinakharinwirot University, Nakhon Nayok, Thailand. These isolates were used to evaluate the analytical specificity of the assay.

A total of 25 DNA samples were prepared from cultured nasal swab specimens collected during routine clinical diagnostic procedures. Bacterial identification and antimicrobial susceptibility testing were performed using the VITEK 2 system (bioMérieux, Hazelwood, MO, USA) at the same clinical laboratory. Genomic DNA was subsequently extracted as described in Section 2.1. The resulting DNA pellets were resuspended in nuclease-free distilled water and stored at −20 °C until further analysis.

All specimens were analyzed using both conventional PCR and recombinase polymerase amplification (RPA) assays. PCR served as the reference method for comparative evaluation of the diagnostic performance of the developed RPA assay, including its sensitivity, specificity, and overall agreement with the established molecular detection method.

### 2.3. Primer and Probe Design

Reference nucleotide sequences representing multiple *S. aureus* and MRSA strains were retrieved from the GenBank database. Sequence comparison and region verification were performed using the NCBI Basic Local Alignment Search Tool (BLAST, web version; (https://blast.ncbi.nlm.nih.gov/Blast.cgi (accessed on 18 November 2017)) to identify target-specific conserved regions within the protein A (*spa*) locus for *S. aureus* (GenBank accession no.M18264.1) and the penicillin-binding protein 2a (*mecA*) gene for MRSA (GenBank accession no.X52593.1). Candidate oligonucleotides were designed in accordance with TwistAmp^®^ nfo assay recommendations, with amplification primers preferentially selected to be approximately 30–35 nucleotides in length and screened to minimize unfavorable secondary structure, primer–primer complementarity, repetitive motifs, and extreme GC content. A short amplicon architecture was favored to enable efficient recombinase polymerase amplification under isothermal conditions.

**Figure 1:**
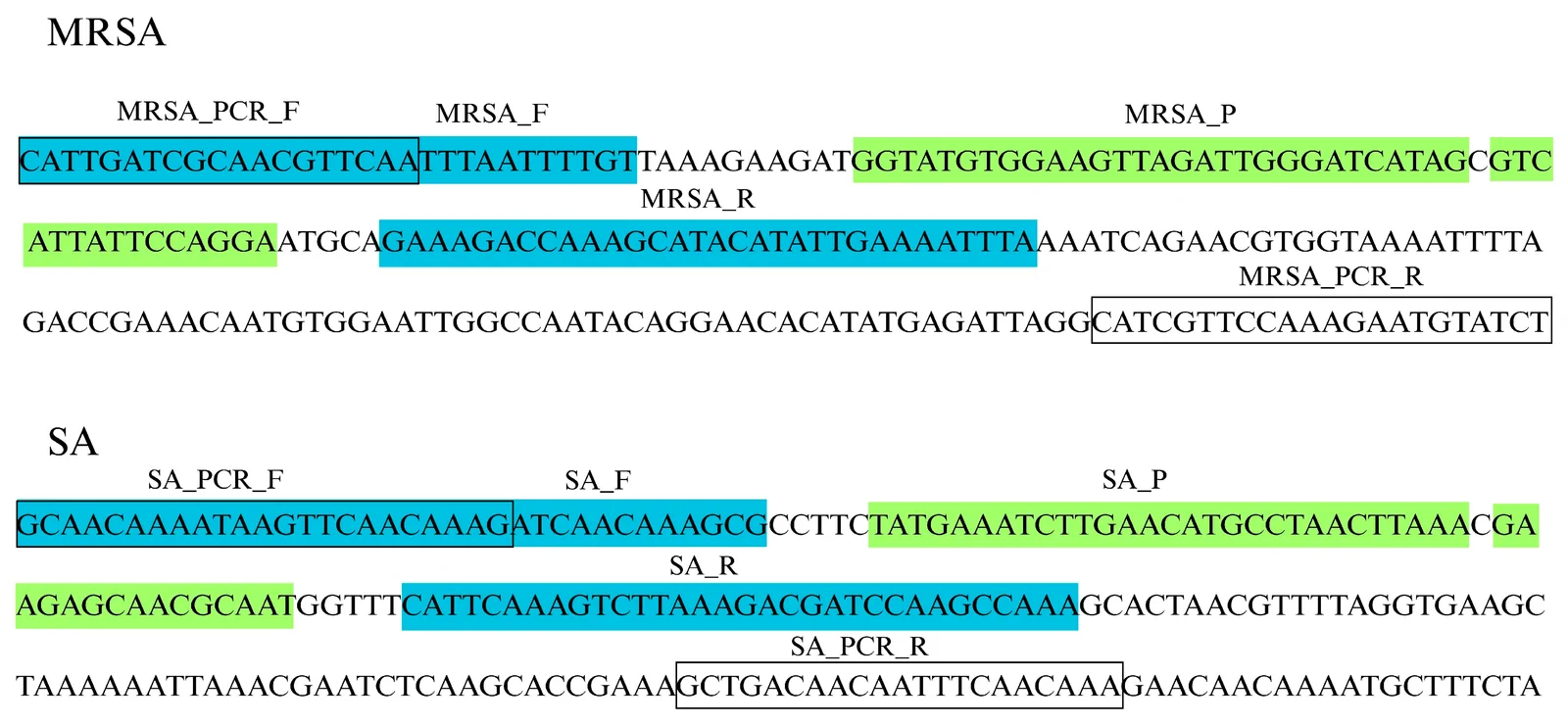
Nucleotide sequences of the *mecA* gene (MRSA) and the *spa* gene (*S. aureus*), illustrating the binding regions of primers and probes used in the duplex RPA–LFD assay and conventional PCR. The RPA primer-binding regions (MRSA_F, MRSA_R, SA_F, and SA_R) are highlighted in blue, whereas the RPA probe-binding regions (MRSA_P and SA_P) are highlighted in green. The conventional PCR primer-binding regions (MRSA_PCR_F, MRSA_PCR_R, SA_PCR_F, and SA_PCR_R) are indicated by black lines and/or outlined boxes, as shown in the figure. The labels indicate the corresponding primer and probe positions within each target sequence.

For lateral-flow detection, internal nfo probes were designed as labeled oligonucleotides containing a 5′ fluorescein isothiocyanate (FITC) reporter, an internal tetrahydrofuran (THF) residue serving as the endonuclease recognition site, and a 3′ C3 spacer to prevent polymerase extension before cleavage. Probe placement was selected to avoid unfavorable overlap with the opposing primer and to preserve target-specific hybridization efficiency. For duplex target discrimination on the lateral flow strip, the MRSA reverse primer carried a 5′ biotin label, whereas the *S. aureus* reverse primer carried a 5′ digoxigenin (DIG) label, thereby enabling the generation of differentially captured dual-labeled amplicons. Multiple candidate primer–probe combinations were considered during assay development, and the final sets were selected empirically based on amplification efficiency, visual detectability of both duplex targets, and absence of nonspecific reactivity. Final primer and probe sequences used for RPA-LFD and conventional PCR are summarized in Table 1, and their genomic locations are shown in Figure 1.

### 2.4. Recombinase Polymerase Amplification (RPA)

Duplex RPA reactions were performed using the TwistAmp^®^ nfo kit (TwistDx, Cambridge, UK) in a final volume of 50 µL. Each reaction contained 29.5 µL rehydration buffer, 2.1 µL of each forward and reverse primer (10 µM), 0.6 µL probe, 14.9 µL nuclease-free water, 1 µL betaine (5 M), and 1 µL template DNA. The reaction mixture was transferred to tubes containing lyophilized enzyme pellets. Magnesium acetate (2.5 µL, 280 mM) was added to initiate amplification, followed by brief centrifugation and mixing. Reactions were incubated at 39 °C for 15 min. Amplification products were analyzed either by agarose gel electrophoresis or by lateral flow detection as described below. Where indicated, products were resolved on 2% agarose gels at 100 V and visualized under UV illumination.

### 2.5. Lateral Flow Dipstick (LFD) Detection

**Figure 2:**
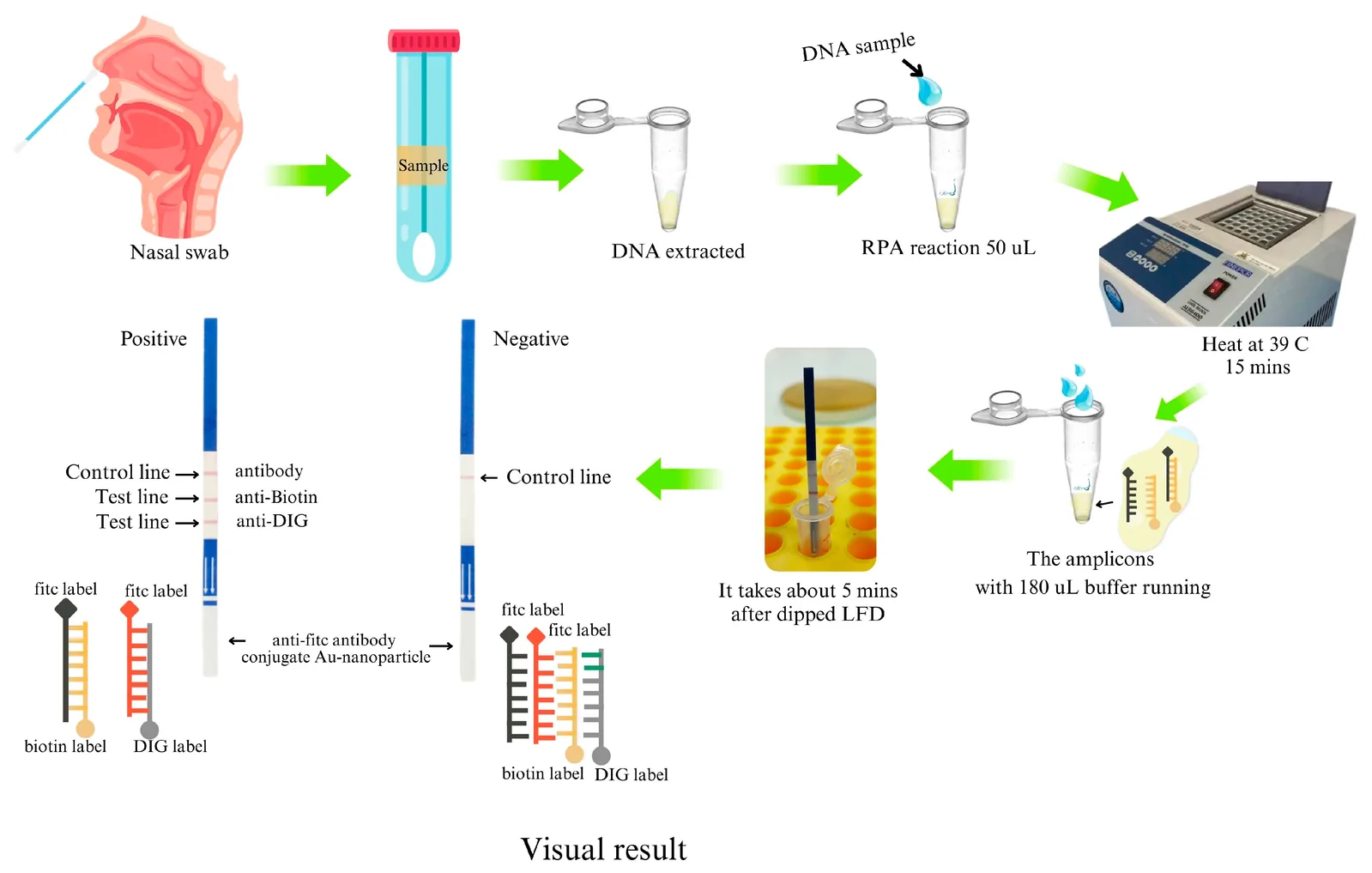
Workflow of duplex RPA coupled with lateral flow dipstick (LFD) detection.

Following amplification, the entire RPA reaction was mixed with 120 µL detection buffer. A customized lateral flow dipstick (K.BIO SCIENCES Co., Ltd., Pathum Thani, Thailand) was inserted directly into the mixture and incubated at room temperature for 5 min (Figure 2). Detection was based on FITC-labelled amplicons forming complexes with gold-conjugated anti-FITC antibodies. Target-specific capture occurred at the corresponding test lines via interaction with immobilized biotin-binding molecules (MRSA) or anti-DIG antibodies (SA), while a separate control line confirmed proper strip function. Results were interpreted visually.

### 2.6. Polymerase Chain Reaction (PCR)

The PCR amplification was conducted using specific primer pairs targeting the *spa* and *mecA* genes (Table 1). The cycling protocol consisted of an initial denaturation at 95 °C for 2 min, followed by 30 cycles of 95 °C for 15 s, 56 °C for 15 s, and 72 °C for 1 min, with a final extension at 72 °C for 5 min. PCR products were analyzed by 2% agarose gel electrophoresis at 100 V and visualized under UV light.

### 2.7. Optimization of Duplex RPA–LFD Conditions

Temperature and incubation time were evaluated to determine suitable reaction parameters. The RPA-LFD assays were performed at discrete temperatures of 30, 35, 40, and 45 °C and incubation periods ranging from 5 to 30 min. The 39 °C operating temperature used in subsequent experiments was selected based on the recommended operating conditions of the TwistAmp^®^ nfo chemistry and was not directly included among the experimentally tested temperatures.

### 2.8. Analytical Sensitivity and Specificity

Ten-fold serial dilutions ranging from 10^6^ to 10^0^ CFU/mL were used for a preliminary analytical sensitivity assessment. Genomic DNA was extracted from each dilution, and 1 µL was used as a template for RPA–LFD and conventional PCR under the conditions described above. Because this dilution series was evaluated in a single experiment without replicate testing around the detection boundary, the lowest concentration producing a visually detectable signal is reported as the observed detection threshold. Analytical specificity was evaluated using genomic DNA extracted from MRSA, *S. pneumoniae*, *K. pneumoniae*, *S. mitis*, *S. mutans*, *S. enterica*, *E. coli*, *M. tuberculosis*, *L. paracasei*, *A. naeslundii*, *A. actinomycetemcomitans*, *P. intermedia*, *P. gingivalis*, *S. aureus*, and *S. epidermidis*. Distilled water was included as a negative control.

### 2.9. Clinical Specimen Analysis

Both RPA-LFD and PCR were used to test all 25 clinical DNA samples under the conditions described above. Results were compared directly to determine concordance. Agreement measures, including positive percent agreement (PPA), negative percent agreement (NPA), and their corresponding 95% confidence intervals (CI), were calculated to describe the concordance of the duplex RPA–LFD assay with conventional PCR and culture methods.

## 3. Results

### 3.1. Optimization of Incubation Time and Temperature for Duplex RPA–LFD

**Figure 3:**
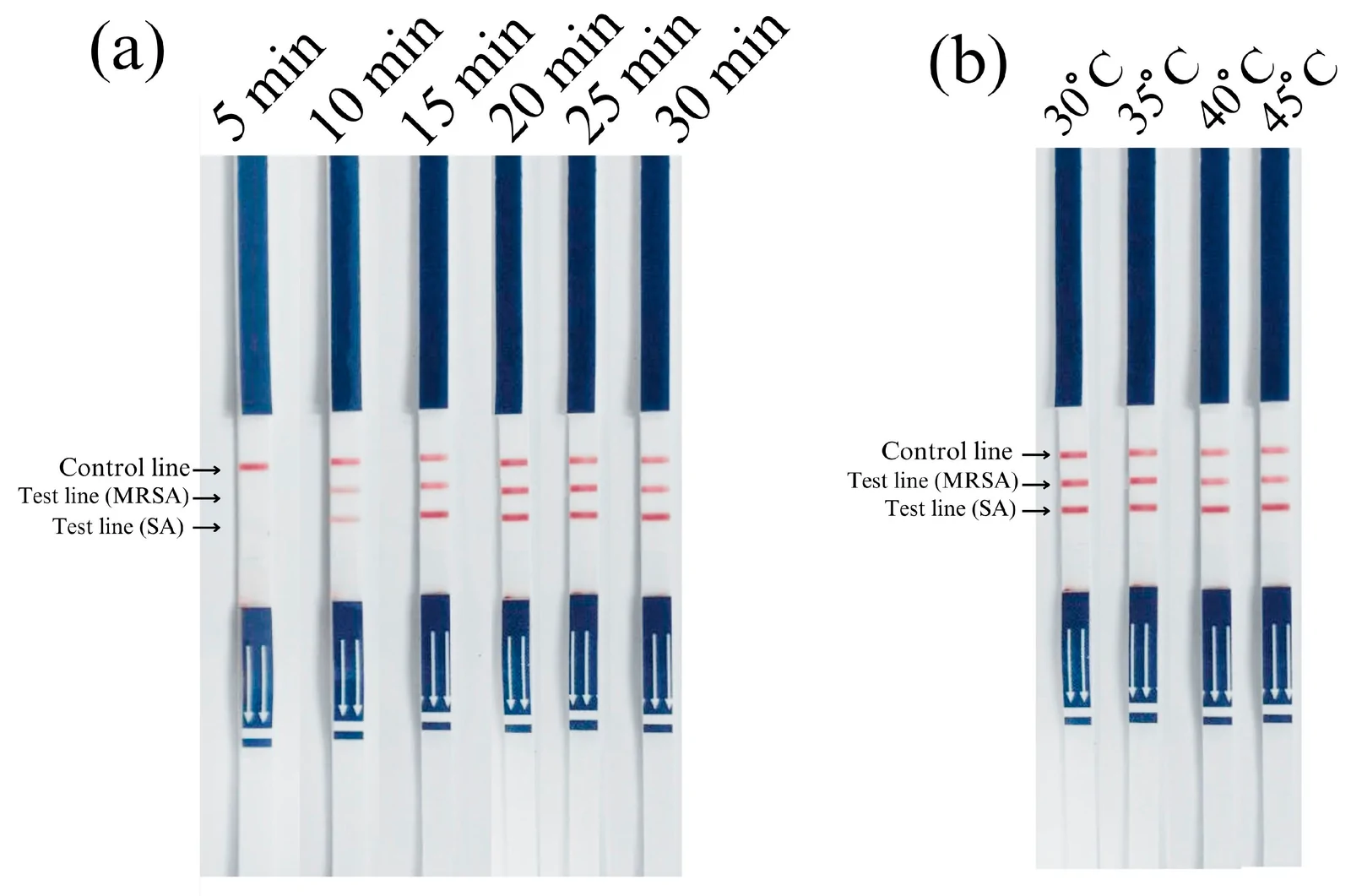
Optimization of incubation time and temperature for duplex RPA–LFD of MRSA and SA. (**a**) Time-course evaluation from 5 to 30 min. (**b**) Temperature optimization performed at discrete temperatures of 30, 35, 40, and 45 °C. SA, *S. aureus*.

The reaction conditions for the duplex RPA–LFD assay were optimized by evaluating the effects of incubation time and temperature using a bacterial input of 10^6^ CFU/mL for both MRSA and *S. aureus*. During time-course analysis, the intensity of the test lines increased progressively between 5 and 30 min (Figure 3a). Faint but visible signals were observed at 5 min, while clearly distinguishable test lines for both targets were observed from 10 min onward.

Reaction temperature was evaluated at discrete points of 30, 35, 40, and 45 °C (Figure 3b). Detectable signals were observed across all tested temperatures, with similar signal intensity observed between 35 and 45 °C. A temperature of 39 °C was selected for subsequent experiments based on the recommended operating conditions of the TwistAmp^®^ nfo chemistry; 39 °C was not directly included among the experimentally tested temperatures. Accordingly, 39 °C was used as the selected operating temperature rather than identified as an experimentally determined temperature optimum, with an incubation time of 15 min used as the standard setting for subsequent duplex RPA–LFD assays.

### 3.2. Preliminary Analytical Sensitivity Assessment

**Figure 4:**
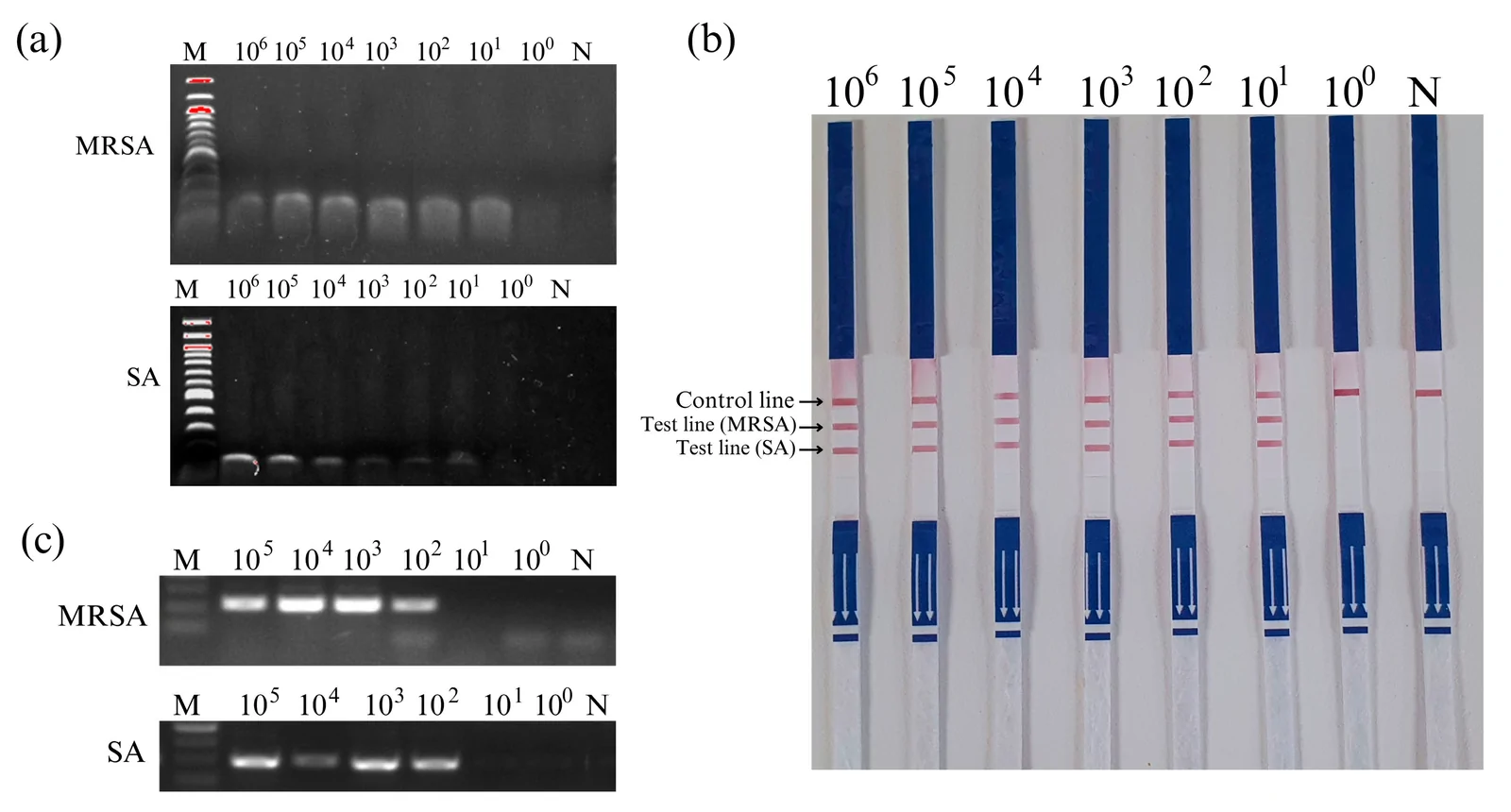
Preliminary analytical sensitivity assessment of duplex RPA–LFD and conventional PCR. (**a**) Agarose gel electrophoresis of RPA products showing visible amplification down to 10^1^ CFU/mL in the tested dilution series. (**b**) Corresponding duplex RPA–LFD results. (**c**) Conventional PCR products showing visible amplification down to 10^2^ CFU/mL under the PCR conditions used in this study. The images represent a single preliminary serial-dilution experiment and were not used to establish a statistically validated analytical detection limit. Lanes 1–7 represent 10-fold serial dilutions from 10^6^ to 10^0^ CFU/mL; lane N represents the negative control (H_2_O). SA, *S. aureus*.

In the preliminary ten-fold serial dilution experiment, visible RPA products were observed down to 10^1^ CFU/mL for both targets (Figure 4a). The corresponding RPA–LFD strips likewise showed visually detectable test lines at 10^1^ CFU/mL (Figure 4b). Under the PCR conditions used in this study, visible amplification products were observed down to 10^2^ CFU/mL (Figure 4c). Thus, in this single experimental comparison, the lowest concentration producing a detectable signal differed by one ten-fold dilution between RPA–LFD and conventional PCR. Because independent replicate testing and additional concentrations around the detection boundary were not performed, these findings are interpreted as preliminary observed detection thresholds and not as statistically validated analytical detection limits.

### 3.3. Analytical Specificity of Duplex RPA-LFD

**Figure 5:**
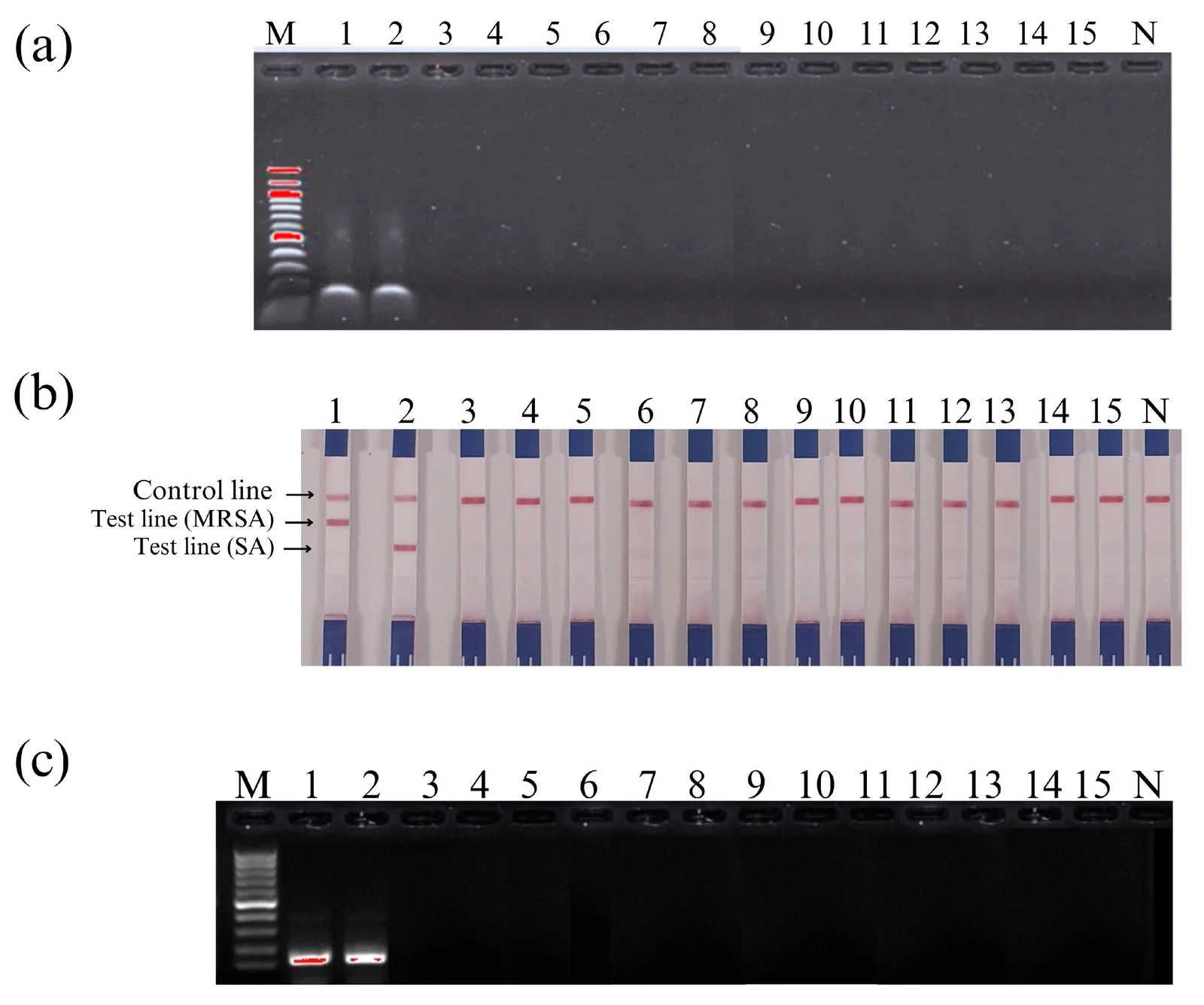
Analytical specificity of the duplex RPA-LFD assay. (**a**) Agarose gel electrophoresis of duplex RPA products. (**b**) Lateral flow dipstick (LFD) results showing target-specific test lines. (**c**) Agarose gel electrophoresis of PCR products. Lane M, 100 bp DNA ladder; lane 1, MRSA; lane 2, SA; lane 3, *S. pneumoniae*; lane 4, *K. pneumoniae*; lane 5, *S. mitis*; lane 6, *S. mutans*; lane 7, *S. enterica*; lane 8, *E. coli*; lane 9, *M. tuberculosis*; lane 10, *L. paracasei*; lane 11, *A. naeslundii*; lane 12, *A. actinomycetemcomitans*; lane 13, *P. intermedia*; lane 14, *P. gingivalis*; lane 15, *S. epidermidis*; N, negative control (H_2_O). SA, *S. aureus*.

Analytical specificity of the duplex RPA–LFD assay was evaluated using genomic DNA derived from MRSA, *S. aureus*, *S. pneumoniae*, *K. pneumoniae*, *S. mitis*, *S. mutans*, *S. enterica*, *E. coli*, *M. tuberculosis*, *L. paracasei*, *A. naeslundii*, *A. actinomycetemcomitans*, *P. intermedia*, *P. gingivalis*, and *S. epidermidis*, together with a no-template water control (Figure 5).

Agarose gel electrophoresis of duplex RPA products produced visible amplification bands exclusively for MRSA (lane 1) and *S. aureus* (lane 2), while no amplification bands were observed for any of the non-target organisms or the negative control (Figure 5a).

Consistent with the gel-based results, the duplex RPA–LFD assay generated three lines for MRSA, corresponding to the control line, MRSA-specific test line (*mecA*), and *S. aureus*-specific test line (*spa*), indicating the simultaneous presence of both targets. In contrast, *S. aureus* alone produced two lines (control line and *spa* test line), reflecting the absence of *mecA*. No test line signals were detected for any of the remaining species or the negative control (Figure 5b).

Conventional PCR produced an identical specificity pattern, with amplification observed only in MRSA and *S. aureus* samples (Figure 5c).

### 3.4. Clinical Specimen Analysis

A total of 25 nasal swab-derived DNA specimens were analyzed in parallel using duplex RPA–LFD, conventional PCR, and culture-based identification (Table 2). Among these specimens, 10 (40%) were identified as MRSA-positive and 19 (76%) as *S. aureus*-positive by both PCR and culture.

**Figure 6:**
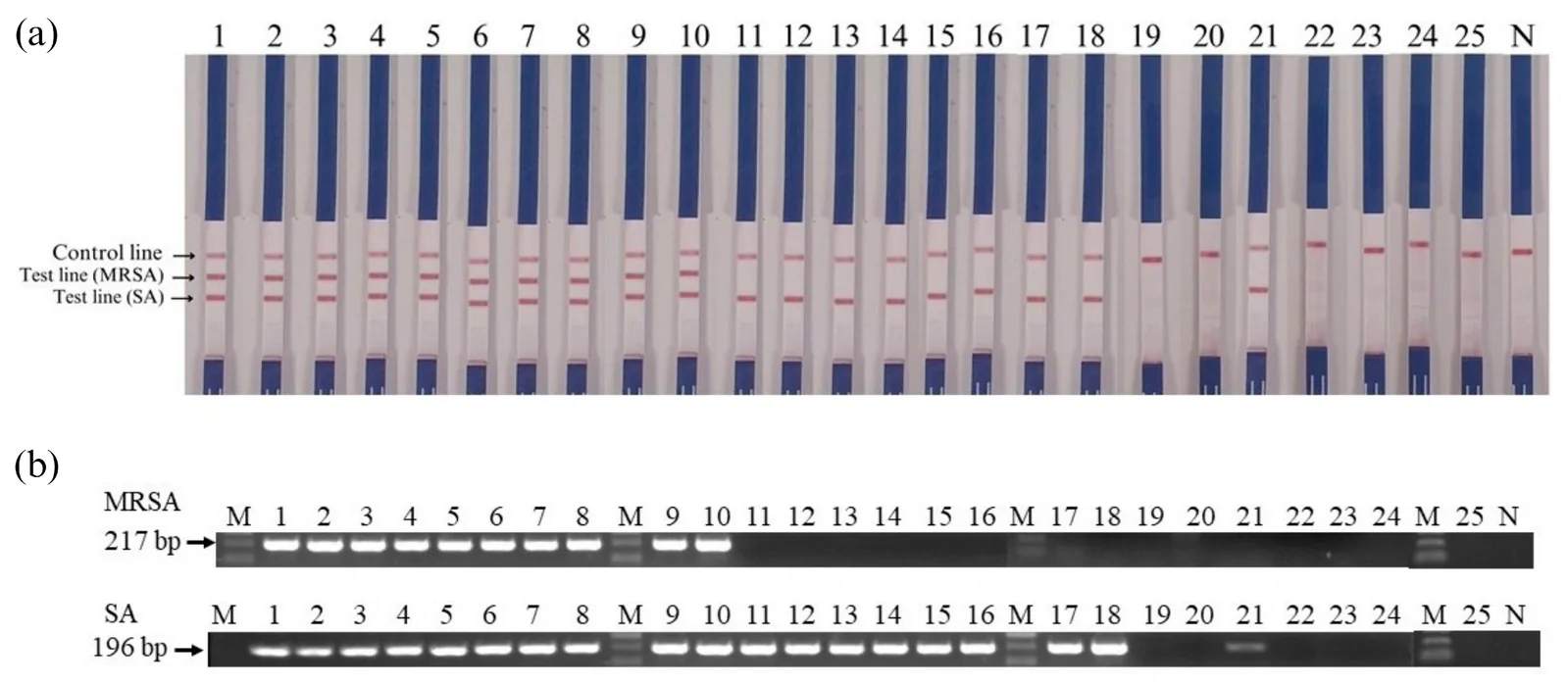
Detection of MRSA and SA in 25 clinical specimens. (**a**) Duplex RPA-LFD results for specimens 1–25 and the no-template control (N). Control line, MRSA test line (*mecA*), and SA test line (*spa*) positions are indicated. (**b**) Agarose gel electrophoresis of duplex PCR products; upper gel shows MRSA-specific amplification (217 bp) and lower gel shows *S. aureus*-specific amplification (196 bp). M, 100 bp DNA ladder; N, negative control (H_2_O); SA, *S. aureus*.

All MRSA-positive specimens generated three visible lines on the lateral flow dipstick (control, *mecA* test line, and *spa* test line), consistent with the presence of both targets. Specimens positive for *S. aureus* alone produced two lines (control and *spa*), whereas specimens negative for both targets showed only the control line (Figure 6a). PCR confirmed MRSA-specific amplification at 217 bp and *S. aureus* amplification at 196 bp in the corresponding positive specimens, with no amplification observed in negative samples (Figure 6b).

Clinical specimens underwent DNA extraction before amplification analysis. The overall concordance between the duplex RPA–LFD assay and conventional PCR was 100% (25/25 specimens). For MRSA detection, both the PPA and NPA of the duplex RPA–LFD assay relative to PCR were 100.0% (95% CI: 72.3–100.0% and 79.6–100.0%, respectively). For *S. aureus* detection, the assay demonstrated a PPA of 100.0% (95% CI: 83.2–100.0%) and an NPA of 100.0% (95% CI: 61.0–100.0%).

## 4. Discussion

This study demonstrates the feasibility of integrating simultaneous detection of the *spa* and *mecA* targets into a single-tube duplex RPA–LFD platform for rapid identification of *S. aureus* and MRSA. Under the selected operating conditions, visible test-line signals were obtained within 15 min at 39 °C, and in the preliminary serial-dilution experiment, visible detection was observed down to 10^1^ CFU/mL under the tested conditions. The ability to generate species identification and methicillin-resistance information within a single isothermal reaction highlights the potential utility of duplex RPA–LFD as a rapid screening approach for MRSA colonization, while further analytical and clinical validation is required.

The selected reaction conditions are consistent with the known operating characteristics of recombinase polymerase amplification. RPA is capable of efficient amplification over a relatively broad temperature range and does not require the repeated thermal cycling necessary for conventional PCR [11,25]. In the present study, detectable signals were observed across all tested temperatures, indicating that amplification performance was maintained despite moderate temperature variation. Such tolerance may be advantageous for decentralized testing environments where precise thermal regulation is not always available. Although comparable signal intensities were obtained between 35 °C and 45 °C, 39 °C was selected for subsequent experiments because it lies within the recommended operating range of the TwistAmp^®^ nfo chemistry; importantly, 39 °C was not directly tested in the temperature optimization experiment. Thus, 39 °C represents the selected operating condition based on the recommended chemistry range rather than an experimentally determined temperature optimum. Likewise, a 15 min incubation period was selected as a practical balance between assay speed and reliable visual interpretation. The simultaneous visual detection of the *spa* and *mecA* test lines under the selected conditions indicate that both targets could be detected concurrently in the tested duplex configuration. However, because duplex and single-plex reactions were not quantitatively compared, the extent of inter-target competition cannot be determined from the present data. This observation is noteworthy because amplification bias can occur in multiplex recombinase-based systems as a consequence of differences in primer–template interactions and competition for shared reaction components [11,14].

In the preliminary dilution experiment, RPA–LFD produced a detectable signal at one ten-fold dilution below that observed with conventional PCR. This finding should not be interpreted as evidence of an intrinsic analytical sensitivity advantage of RPA over PCR. The two assays used different primer sets, amplification chemistries, reaction conditions, and endpoint detection formats, each of which may influence the observed detection threshold. Accordingly, the comparison is presented only as an assay-specific observation under the experimental conditions used in this study. Replicate testing around the detection boundary would be required for a rigorous comparison of analytical sensitivity. Although both spa and *mecA* signals were visually detectable under the selected duplex conditions, the present study did not quantitatively compare test-line intensities, directly compare duplex with single-plex reactions, or evaluate highly unequal target concentrations. Therefore, the current data demonstrate concurrent qualitative detection rather than quantitatively balanced amplification. Further experiments are required to determine the extent of inter-target competition, particularly when one target is present near its detection threshold.

No cross-reactivity was observed among the non-target organisms included in the specificity panel, indicating that the selected primer–probe combinations were highly selective under the conditions tested. However, an important limitation is that methicillin-resistant coagulase-negative staphylococci (MRCoNS) were not included in the current evaluation. Because the *mecA* gene is distributed among multiple staphylococcal species, particularly coagulase-negative staphylococci that commonly colonize human skin and the nasal cavity [4,26], future studies should include methicillin-resistant *Staphylococcus epidermidis* and related species to further assess the specificity of the *mecA* detection line under clinically relevant conditions. An additional limitation arises in mixed staphylococcal populations. Because the assay detects spa and *mecA* as separate molecular targets, a specimen containing methicillin-susceptible *S. aureus* together with a mecA-positive coagulase-negative Staphylococcus could theoretically generate both test lines and mimic an MRSA-positive result. This scenario was not experimentally evaluated in the present study and should be specifically investigated using defined mixed cultures or DNA mixtures in future validation studies.

The choice of *spa* as the species-specific target warrants consideration. Previous molecular assays for MRSA detection have frequently combined *nuc* and *mecA*, including a recently reported duplex RPA–LFD assay developed for blood-culture specimens [27]. Representative recombinase-based assays for MRSA detection are compared in Supplementary Table S1. Previous studies have demonstrated rapid detection of methicillin-resistant staphylococci using mecA-targeted RPA–LFD and MRSA identification using nuc- and mecA-targeted RPA–LF or duplex ERA–LFA configurations. In contrast, the present study evaluates a spa/mecA target combination within a single duplex RPA–LFD reaction and applies this configuration to nasal swab-derived DNA specimens. Thus, the contribution of the present study lies primarily in the specific target combination and duplex assay architecture rather than in the general use of recombinase-based amplification or lateral-flow detection. In the present study, *spa* was selected because MRSA identification was intended to rely on simultaneous detection of a species-specific marker and a methicillin-resistance determinant. The polymorphic X-region of *spa* has been widely used for molecular typing and epidemiological characterization of *S. aureus* and remains an established target for species-level identification [28,29]. Accordingly, concurrent detection of *spa* and *mecA* provides species- and resistance-associated information within a single assay. However, because the two targets are detected independently, their co-detection does not establish that both determinants originate from the same bacterial cell. This limitation is particularly relevant in mixed staphylococcal populations. The optimization, preliminary analytical sensitivity, and analytical specificity experiments presented in Figure 3, Figure 4 and Figure 5 were conducted as single-run exploratory assessments. Consequently, between-run reproducibility and variability could not be statistically evaluated. Lateral-flow results were interpreted qualitatively by visual inspection, and quantitative test-line intensity analysis was not performed. A further limitation is that the optimization, analytical sensitivity, and specificity experiments were not repeated as independent experimental runs. Consequently, the present study does not establish inter-assay reproducibility or quantitative variability of lateral-flow signal intensity. These parameters should be addressed in subsequent analytical validation using independent replicate experiments and standardized quantitative strip analysis.

Primer design is a critical component of any nucleic acid amplification assay, particularly in duplex configurations where amplification balance must be maintained between multiple targets [11,14]. In the present study, conserved regions of the *spa* and *mecA* genes were identified through sequence alignment before primer and probe design. The resulting oligonucleotides were selected to minimize secondary structure formation, reduce primer–primer interactions, and maintain comparable primer lengths and GC contents [11,14]. However, the assay was not evaluated using a large collection of genotyped MRSA isolates representing diverse clonal complexes and SCCmec lineages. Consequently, the breadth of sequence coverage across the genetic diversity of contemporary MRSA populations remains incompletely characterized. Future investigations should therefore include well-characterized strain collections representing diverse epidemiologically relevant lineages to further establish the robustness and universality of the assay. In addition, the present design targets *mecA* only and would not detect MRSA strains carrying alternative resistance determinants such as *mecC* [4,26].

A preliminary evaluation of 25 nasal swab-derived DNA specimens demonstrated strong agreement between duplex RPA–LFD and conventional PCR. Both the positive percent agreement (PPA) and negative percent agreement (NPA) reached 100% for MRSA detection, and *S. aureus* identification likewise demonstrated a PPA of 100% and an NPA of 100%.

From a clinical perspective, rapid identification of MRSA nasal colonization has become increasingly relevant within antimicrobial stewardship programs. Molecular MRSA nasal screening has been associated with earlier discontinuation of empiric anti-MRSA therapy, reduced unnecessary vancomycin exposure, and support for antimicrobial de-escalation strategies in patients with suspected respiratory infections [22,30,31]. Although the present study was not designed to evaluate clinical outcomes, the operational characteristics of the duplex RPA–LFD platform—including rapid turnaround time, isothermal amplification, and instrument-free visual interpretation—are compatible with screening workflows where timely decision-making is desirable. Nevertheless, the current protocol still relies on laboratory-based DNA extraction procedures involving centrifugation and heat-treatment steps. Integration with simplified extraction methods or portable sample-to-result workflows would therefore be necessary before routine implementation in community-based or resource-limited settings could be realistically considered. Accordingly, the 15 min reaction time reported here refers only to RPA and does not represent the total sample-to-result turnaround time.

Several limitations of this study should be acknowledged. First, the clinical sample size (n = 25) was insufficient for reliable estimation of diagnostic sensitivity, specificity, or predictive values, and the present findings should therefore be interpreted as preliminary concordance data pending validation in larger cohorts, consistent with STARD 2015 reporting recommendations [32]. Second, comprehensive phenotypic antimicrobial susceptibility testing was not performed for all clinical specimens, introducing uncertainty regarding confirmation of functional methicillin resistance in every mecA-positive isolate. Third, the specificity panel did not include MRCoNS, and inclusion of such strains will be necessary to further evaluate discrimination at the *mecA* detection line. Fourth, the assay detects *mecA* but does not characterize SCCmec structure or identify alternative resistance determinants such as *mecC*. Finally, the current duplex format does not incorporate an internal amplification control, preventing differentiation between true-negative and inhibition-associated false-negative results. Future work should therefore focus on incorporating internal controls, evaluating genetically diverse MRSA lineages, expanding validation with larger clinical cohorts, and developing simplified sample-preparation strategies to facilitate practical implementation outside conventional laboratory settings.

## 5. Conclusions

This study demonstrates the feasibility of configuring duplex RPA–LFD for concurrent qualitative detection of the S. aureus-associated spa locus and the methicillin-resistance determinant *mecA* within a single isothermal reaction. Preliminary analytical experiments demonstrated visible detection of both targets across the tested dilution series, while evaluation of 25 nasal swab-derived DNA specimens showed complete concordance with PCR within this limited sample set. However, the analytical experiments were exploratory and require independent replicate validation, quantitative assessment of duplex performance, evaluation against mecA-positive coagulase-negative staphylococci and mixed populations, and validation in a larger clinical cohort. The present platform should therefore be regarded as a technical framework for further development rather than a fully validated point-of-care diagnostic assay.

## Figures and Tables

**Table 1:** Primer and probe sequences targeting the *spa* and *mecA* genes used for duplex RPA–LFD and conventional PCR.

Name	Sequence 5′–3′	GC Content (%)	Length (nt)	Melting Temperature (Tm; °C)	Amplicon Size (bp)
SA-F	gCAACAAAATAAgTTCAACAAAgATCAACAAAgCg	34.3	35	59.7	122
SA-R	DIG-TTTggCTTggATCgTCTTTAAgACTTTgAATg	37.5	32	59.3	122
SA-P	FITCTATgAAATCTTgAACATgCCTAACTTAAAXgAAgAgCAACgCAAT-C3 spacer	34.1	44	77.0	122
SA_PCR_F	gCAACAAAATAAgTTCAACAAAg	30.4	23	59.3	196
SA_PCR_R	TTTgTTgAAATTgTTgTCAgC	33.3	21	60.2	196
MRSA-F	CATTgATCgCAACgTTCAATTTAATTTTgT	30.0	30	70.6	122
MRSA-R	Biotin-TTAAATTTTCAATATgTATgCTTTggTCTTTC	25.0	32	66.0	122
MRSA-P	FITCggTATgTggAAgTTAgATTgggATCATAgXgTCATTATTCCAggA-C3 spacer	40.9	44	77.9	122
MRSA_PCR_F	CATTgATCgCAACgTTCAA	42.1	19	63.4	217
MRSA_PCR_R	AgATACATTCTTTggAACgATg	36.4	22	59.5	217

**Note:** F, forward primer; R, reverse primer; P, probe; DIG, digoxigenin; FITC, fluorescein isothiocyanate; X, a tetrahydrofuran (THF) residue; C3, C3 spacer (3′ blocking group).

**Table 2:** Clinical detection results for MRSA and *S. aureus* using duplex RPA–LFD, PCR, and culture methods in 25 nasal swab specimens.

Target/Assay	Positive	Negative	PPA (95% CI)	NPA (95% CI)
MRSA				
RPA–LFD	10	15	100.00%	100.00%
PCR/Culture	10	15	(72.3–100.0%)	(79.6–100.0%)
*S. aureus* (SA)				
RPA–LFD	19	6	100.00%	100.00%
PCR/Culture	19	6	(83.2–100.0%)	(61.0–100.0%)

## Data Availability

All data supporting the findings of this study are available within the article. Any additional raw data is available from the corresponding author upon reasonable request.

## Supplementary Materials

The following supporting information can be downloaded at https://www.mdpi.com/article/10.3390/diagnostics16203295/s1, Table S1. Comparison of the present duplex RPA–LFD platform with representative recombinase-based assays for detection of methicillin-resistant staphylococci and MRSA. References [33,34] are cited in the Supplementary Materials.

## Abbreviations

CFU, colony-forming unit; LFD, lateral flow dipstick; MRSA, methicillin-resistant *Staphylococcus aureus*; PCR, polymerase chain reaction; PBP2a, penicillin-binding protein 2a; RPA, recombinase polymerase amplification; SA, *Staphylococcus aureus*.
