# Supplementary material for: A Duplex Isothermal RPA–Lateral Flow Platform for Concurrent Detection of Staphylococcus Aureus and mecA-Mediated Methicillin Resistance
Source: Diagnostics (Basel). 2026 Oct 10;16(20):3295. doi: 10.3390/diagnostics16203295 (PMC13703673; doi:10.3390/diagnostics16203295)
Supplement: Supplementary file 1 [file diagnostics-16-03295-s001.zip › diagnostics-4606402-supplementary.pdf]

Supplementary Table S1. Comparison of the present duplex RPA–LFD platform with representative recombinase-based assays for detection of methicillin-resistant staphylococci and MRSA

Study	Amplification / detection	Molecular target(s)	Assay configuration	Reaction conditions	Analytical detection limit*	Clinical evaluation	Key distinction from the present study
[1]	RPA–LFD	*mecA*	Single-target RPA–LFD for methicillin-resistant *Staphylococcus* spp.	45 °C, 20 min RPA + 5 min LFD	Reported to be comparable with PCR	Positive blood cultures; 92.1% sensitivity and 100% specificity for methicillin-resistant staphylococci	Detects *mecA* alone and therefore identifies methicillin-resistant staphylococci rather than simultaneously establishing an *S. aureus*-associated marker and *mecA* status.
[2]	RPA–LF	*nuc* + *mecA*	Separate *nuc*- and *mecA*-RPA–LF assays used for MRSA identification	45 °C, 20 min RPA + 5 min LF	10 CFU/reaction for both *nuc* and *mecA* by RPA-LF	60 positive blood-culture samples; *nuc*: 93.3% sensitivity, 100% specificity; *mecA*: 100% sensitivity, 97.7% specificity	Direct testing of positive blood cultures without DNA purification; *nuc* and *mecA* were evaluated using target-specific RPA-LF assays rather than the present *spa*/*mecA* duplex target combination.
[3]	Duplex ERA–LFA	*nuc* + *mecA*	Duplex enzymatic recombinase amplification coupled with lateral-flow detection	Isothermal ERA–LFA	1 CFU/reaction (*nuc*); 10 CFU/reaction (*mecA*)	46 clinical isolates plus 57 positive blood-culture samples	Uses a duplex *nuc*/*mecA* ERA–LFA architecture and positive blood-culture specimens; represents a particularly relevant recent comparator to

Study	Amplification / detection	Molecular target(s)	Assay configuration	Reaction conditions	Analytical detection limit*	Clinical evaluation	Key distinction from the present study
							the present duplex assay.
Present study	Duplex RPA–LFD	spa* + *mecA	Concurrent detection of the *S. aureus*-associated *spa* locus and *mecA* within a single RPA reaction with visually distinguishable LFD test lines	39 °C, 15 min RPA	Visible detection to 10^1 CFU/mL in a single preliminary serial-dilution experiment; not a statistically validated LOD	25 nasal swab-derived DNA specimens; 100% concordance with PCR in this limited sample set; MRSA PPA/NPA 100% with 95% CIs of 72.3–100% and 79.6–100%, respectively	Uses the *spa*/*mecA* target combination in a single duplex RPA–LFD configuration and evaluates its feasibility with nasal swab-derived DNA; analytical and clinical findings remain preliminary.

* Detection-limit values should not be directly compared across studies because specimen preparation, concentration units, amplification chemistry, primer/probe designs, reaction conditions, and endpoint detection methods differ.

References

1. Srisrattakarn, A.; Tippayawat, P.; Chanawong, A.; Tavichakorntrakool, R.; Daduang, J.; Wonglakorn, L.; Sooksongsoontorn, P.; Lulitanond, A. Direct detection of methicillin-resistant in *Staphylococcus* spp. in positive blood culture by isothermal recombinase polymerase amplification combined with lateral flow dipstick assay. *World J. Microbiol. Biotechnol.* **2020**, *36*, 162. https://doi.org/10.1007/s11274-020-02938-8.
2. Srisrattakarn, A.; Panpru, P.; Tippayawat, P.; Chanawong, A.; Tavichakorntrakool, R.; Daduang, J.; Wonglakorn, L.; Lulitanond, A. Rapid detection of methicillin-resistant *Staphylococcus aureus* in positive blood-cultures by recombinase polymerase amplification combined with lateral flow strip. *PLoS ONE* **2022**, *17*, e0270686. https://doi.org/10.1371/journal.pone.0270686.
3. Srisrattakarn, A.; Chanawong, A.; Sripichai, O.; Rujidamp, J.; Maneenet, N.; Onsapiw, N.; Bunleuhan, T.; Lulitanond, A. Duplex Enzymatic Recombinase Amplification Assay Coupled with Lateral Flow Dipstick for Rapid Identification of Methicillin-Resistant *Staphylococcus aureus*. *ACS Infect. Dis.* **2025**, *11*, 3188–3195. https://doi.org/10.1021/acsinfecdis.5c00576.
